# Supplementary material for: The Role of Passion and Achievement Goals in Optimal Functioning in Sports
Source: Int J Environ Res Public Health. 2021 Aug 28;18(17):9023. doi: 10.3390/ijerph18179023 (PMC8431141; doi:10.3390/ijerph18179023)
Supplement: Supplementary file 1 [file ijerph-18-09023-s001.zip › ijerph-1342672-supplementary.pdf]

International Journal of
Environmental Research and Public Health

MDPI

The Role of Passion and Achievement Goals in Optimal Functioning in Sports - Supplementary

Measured items

Passion Scale

While thinking about your sport, please indicate your level of agreement with each of the following items, from 1= do not agree at all to 7 = Agree completely.

	1	2	3	4	5	6	7
1) I am completely taken with synchronized swimming/water polo.							
2) I cannot live without synchronized swimming/water polo.							
3) Synchronized swimming/water polo allows me to live memorable experiences.							
4) I am emotionally dependent on synchronized swimming/water polo.							
5) My mood depends on me being able to do synchronized swimming/water polo.							
6) Synchronized swimming/water polo is in harmony with other activities in my life.							
7) I have a tough time controlling my need to do synchronized swimming/water polo.							
8) The new things I discover with synchronized swimming/water polo allow me to appreciate it even more.							
9) I have almost an obsessive feeling for synchronized swimming/water polo.							
10) I cannot imagine my life without synchronized swimming/water polo.							
11) For me synchronized swimming/water polo is a passion, that I can still manage to control.							
12) Synchronized swimming/water polo reflects the qualities I like about myself.							
13) Synchronized swimming/water polo allows me to live a variety of experiences.							

14) The urge is so strong. I can't help myself from doing synchronized swimming/water polo.							
15) I spend a lot of time doing it.							
16) I like it a lot.							
17) It is important to me.							
18) It is a passion for me.							

Achievement Goals Questionnaire

Using the 1-7 scale below, indicate the extent to which you believe each item to be true for you.

	1	2	3	4	5	6	7
1) It is important for me to develop my skills as thoroughly as possible this season.							
2) My goal this season is to avoid swimming badly.							
3) It is important for me to do well compared to other swimmers this season.							
4) I want to develop my skills as much as possible this season.							
5) My fear of doing poorly this season is often what motivates me.							
6) I want to swim at the top of my ability by the end of the season.							
7) It is important to me to swim better than other swimmers.							
8) I just want to avoid swimming badly this season.							
9) I hope to have improved and broadened my skill set by the end of the season.							
10) I want to swim well this season so that I can show my skills to my friends, family, coaches, or others.							
11) I often ask myself, "What will happen if I don't swim well this season?"							
12) In a competitive network like this, I prefer to swim against swimmers who push me to develop my skills further.							
13) This season, I am trying to demonstrate my skills compared to other swimmers.							

14) My goal this season is to swim better than most other swimmers.							
15) In a competitive network like this, I prefer to swim against talented swimmers, even if it means I must work harder when I swim against them.							
16) I am motivated to surpass other swimmers this season.							
17) The possibility of swimming poorly this season worries me.							
18) I'm afraid that if I don't swim well, others will think I don't have much talent.							

Coach-athlete relationship

Below are five statements that you may agree or disagree with. Using the 1-7 scale below, identify to which level you have felt that way since the beginning of your competitive season.

I have felt ….

	**1**	**2**	**3**	**4**	**5**	**6**	**7**
1) …supported **by my coach**.							
2) …trust **in my coach**.							
3) …understood **by my coach**.							
4) …listened to **by my coach**.							
5) …valued **by my coach**.							

Performance

Using the scale of 1 to 7, indicate the extent to which you agree with the statements below.

	**1**	**2**	**3**	**4**	**5**	**6**	**7**
1) I feel efficient in the different aspects of synchronized swimming/water polo.							
2) I feel competent in synchronized swimming/water polo.							
3) Overall, I feel I am good at synchronized swimming/water polo.							
4) I feel I have many skills in synchronized swimming/water polo.							
5) I expect to do well this season.							
6) I think I will swim well this season.							
7) I get the most out of my talent and my skills.							
8) I am confident that I can swim							

well.							
9) I tend to swim better under pressure because I think better.							
10) The more pressure there is during my routine, the more I like it.							
11) For me, situations where there is pressure are challenges that I enjoy.							
12) I make fewer mistakes when there is pressure because I focus better.							

Satisfaction With Life Scale

Below are five statements that you may agree or disagree with. Using the 1-7 scale below, indicate your agreement with each item by placing the appropriate number on the line preceding that item. Please be open and honest in your responding.

	1	2	3	4	5	6	7
1) In most ways my life is close to my ideal.							
2) The conditions of my life are excellent.							
3) I am satisfied with my life.							
4) So far, I have gotten the important things I want in life.							
5) If I could live my life over, I would change almost nothing.							

Intentions to continue in sport

Using the scale of 1 to 7, indicate the extent to which you agree with the statements below.

In the future ...

	1	2	3	4	5	6	7
1) ...I will continue to do synchronized swimming/water polo.							
2) ... I will spend more time doing synchronized swimming/water polo.							
3) ... I intend to make synchronized swimming/water polo a career.							
4) ... I intend to give up synchronized swimming/water polo.							
